# Reducing muscle fatigue during transcutaneous neuromuscular electrical stimulation by spatially and sequentially distributing electrical stimulation sources

**DOI:** 10.1007/s00421-013-2807-4

**Published:** 2014-01-05

**Authors:** Dimitry G. Sayenko, Robert Nguyen, Milos R. Popovic, Kei Masani

**Affiliations:** 1 Department of Neurological Surgery, University of Louisville Frazier Rehab Institute, 220 Abraham Flexner Way, Ste. 1506, Louisville, KY 40202 USA; 2 Automatic Control Laboratory, ETH Zurich, Physikstrasse 3, 8092 Zurich, Switzerland; 3 Institute of Biomaterials and Biomedical Engineering, University of Toronto, 164 College Street, Toronto, ON M5S 3G9 Canada; 4 Rehabilitation Engineering Laboratory Lyndhurst Centre, Toronto Rehabilitation Institute - University Health Network, 520 Sutherland Drive, Toronto, ON M4G 3V9 Canada

**Keywords:** Electromyography, Muscle contractile properties, Muscle fatigue, Neuromuscular electrical stimulation

## Abstract

**Purpose:**

A critical limitation with transcutaneous neuromuscular electrical stimulation is the rapid onset of muscle fatigue. We have previously demonstrated that spatially distributed sequential stimulation (SDSS) shows a drastically greater fatigue-reducing ability compared to a single active electrode stimulation (SES). The purposes of this study were to investigate (1) the fatigue-reducing ability of SDSS in more detail focusing on the muscle contractile properties and (2) the mechanism of this effect using array-arranged electromyogram (EMG).

**Methods:**

SDSS was delivered through four active electrodes applied to the plantarflexors, sending a stimulation pulse to each electrode one after another with 90° phase shift between successive electrodes. In the first experiment, the amount of exerted ankle torque and the muscle contractile properties were investigated during a 3 min fatiguing stimulation. In the second experiment, muscle twitch potentials with SDSS and SES stimulation electrode setups were compared using the array-arranged EMG.

**Results:**

The results demonstrated negligible torque decay during SDSS in contrast to considerable torque decay during SES. Moreover, small changes in the muscle contractile properties during the fatiguing stimulation using SDSS were observed, while slowing of muscle contraction and relaxation was observed during SES. Further, the amplitude of the M-waves at each muscle portion was dependent on the location of the stimulation electrodes during SDSS.

**Conclusion:**

We conclude that SDSS is more effective in reducing muscle fatigue compared to SES, and the reason is that different sets of muscle fibers are activated alternatively by different electrodes.

## Introduction

Transcutaneous neuromuscular electrical stimulation (TNMES) is a method to electrically activate muscle fibers by applying stimulation electrodes on the skin above muscles and stimulating branches of motor nerve. TNMES is used to promote physiological and functional improvement in paralyzed limbs (Ragnarsson 2008; Sheffler and Chae 2007; Peckham and Knutson 2005) and counteract musculoskeletal atrophy (Bergquist et al. 2011; Dudley-Javoroski and Shields 2008). Without normal use, paralyzed muscle rapidly atrophies, creating a catabolic state, poor cosmesis, and increased risk for secondary complications (Shields and Cook 1992; Dudley-Javoroski and Shields 2008; Garber and Krouskop 1982; Chantraine et al. 1986; Merli et al. 1993), which ultimately can be life threatening (Shields and Dudley-Javoroski 2003, 2006). While TNMES has succeeded in assisting individuals with neuromuscular disorders, a critical limitation with this rehabilitative approach is the rapid onset of muscle fatigue during repeated contractions (Bickel et al. 2011; Shields and Dudley-Javoroski 2006; Mizrahi et al. 1997), which results in muscle force decay and slowing of muscle contractile properties (Jones 2010; Enoka and Stuart 1992).

The increased fatigability with TNMES is thought by some researchers to reflect a reversal of the size principle of recruitment (Henneman et al. 1965a, b), when larger axons that innervate the more easily fatigable fibers are recruited at low stimulus magnitudes and the smaller axons follow with increased stimulation levels (Peckham and Knutson 2005; Sheffler and Chae 2007; Bickel et al. 2011). Another plausible explanation is that voluntary contraction allows work being shared between different motor units of the same muscle (Bajd et al. 1999; Bickel et al. 2011), whereas conventional TNMES does not permit alterations in recruitment of motor units because all parameters remain fixed during the bout (Bickel et al. 2011). Thus, in stimulated muscles, a synchronized and massive fiber contraction replaces the normal physiological mechanism of motor unit recruitment and firing rate regulation (De Luca 1984). Furthermore, in individuals with neuromuscular disorders, fatigue develops within the motor unit and is associated with such factors as depletion of substances, accumulation of catabolites, and problems in excitation–contraction coupling (Biering-Sorensen et al. 2009; Pelletier and Hicks 2009; Shields 1995). Due to these major factors, the paralyzed muscles show greater fatigability than healthy muscles (Gerrits et al. 1999, 2003; Lenman et al. 1989; Thomas 1997a, b; Shields 1995), thus further compounding the problem of muscle fatigue during TNMES. Consequently, developing means to counter force loss during electrical stimulation has received much interest (Gauthier et al. 1992; Stein et al. 1992; Binder-Macleod and McLaughlin 1997; Riess and Abbas 2001).

Because synchronous activation of an entire muscle is one of the fundamental causes of rapid muscle fatigue during TNMES, it is logical to consider an approach where activating several subcomponents separately is used to reduce muscle fatigue. To achieve this indirectly, early attempts have included stimulation with stochastically modulated parameters such as pulse frequency and amplitude (Graham et al. 2006; Thrasher et al. 2005; Graupe et al. 2000). The results from these studies are not consistent: one study showed that stochastic modulation decreases fatigue (Graupe et al. 2000) and the other two studies showed no significant difference (Graham et al. 2006; Thrasher et al. 2005). In addition, reduced fatigue has been demonstrated using more direct approaches of separately activating subcompartments of muscles in nerve stimulation. This was achieved using multiple electrodes with each activating a different set of nerve fibers and stimulation pulses of relatively low frequencies were sent to each electrode, one after another, resulting in a fused response. This type of spatially distributed and sequentially applied stimulation is referred to as ‘sequential stimulation’ (Nguyen et al. 2011). Fatigue was reduced with such stimulation in animal experimental models using spinal stimulation (Petrofsky 1978, 1979; Mushahwar and Horch 1997), intrafascicular stimulation (McDonnall et al. 2004; Yoshida and Horch 1993), interfascicular stimulation (Thomsen and Veltink 1997), epineural stimulation (Petrofsky 1979), and intramuscular stimulation (Lau et al. 1995; Zonnevijlle et al. 2000; Lau et al. 2007).

To date, observations on this stimulation method in human subjects are limited to only a few studies (Malesevic et al. 2010; Pournezam et al. 1988) including a case report from our group (Nguyen et al. 2011). Pournezam et al. (1988) applied sequential stimulation to three knee extensor muscles in two individuals using three active surface electrodes distributed over these muscles. Malesevic et al. (2010) investigated fatigue reduction using sequential stimulation of the knee extensor muscles through four active surface electrodes distributed over quadriceps as compared to one active electrode. In these two studies (Malesevic et al. 2010; Pournezam et al. 1988), electrodes were spaced far apart, each of which intentionally targeted one motor point of three separate knee extensor synergists rather than allowing different, potentially overlapping, sets of motor units to be activated. Our recent pilot study with one individual with spinal cord injury (Nguyen et al. 2011) was unique in the sense that multiple active surface electrodes providing the interleaved stimulation were collocated at the same site and over the same area as during stimulation with one active electrode targeting a single motor point of a relatively small muscle, as opposed to the previously reported widely distributed electrode setup over the large muscles. We termed such stimulation over multiple active electrodes as spatially distributed sequential stimulation (SDSS). Through four active electrodes, SDSS was delivered by sending a stimulation pulse to each electrode one after another with 90° phase shift between successive electrodes. Single electrode stimulation (SES) was delivered through one active electrode for comparison. We demonstrated that the fatigue resistance improved almost twice in SDSS compared to SES. We hypothesize the effectiveness of the SDSS might be explained by different sets of muscle fibers being activated by different electrodes, and, therefore, the increased time between subsequent activation of motor units allows their greater recovery. However, verification of the obtained result with a larger number of subjects is required and the mechanism of this fatigue reduction is yet to be explored.

The first purpose of this study was to investigate the fatigue-reducing ability of SDSS in more detail, in particular, focusing on the muscle contractile properties and on a larger group of subjects (Experiment 1). The second purpose of this study was to investigate the mechanism of the fatigue-reducing effects of SDSS using array-arranged electromyogram (EMG), by measuring EMG in medial, median, and lateral portions of the soleus muscle in response to single stimuli delivered using SDSS and SES (Experiment 2).

## Materials and methods

### Experiment 1

#### Subjects

Fifteen able-bodied participants (ten male, five female) aged 25.1 ± 6.3 years (mean ± SD), with height of 170.3 ± 6.8 cm and weight of 64.4 ± 6.3 kg, participated in the study. None of the participants had any known history of neurological disorders. Each participant gave written informed consent to the experimental procedure. This study was approved by the local ethics committee in accordance with the Declaration of Helsinki on the use of human subjects in experiments.

#### Transcutaneous neuromuscular electrical stimulation

**Fig. 1:**
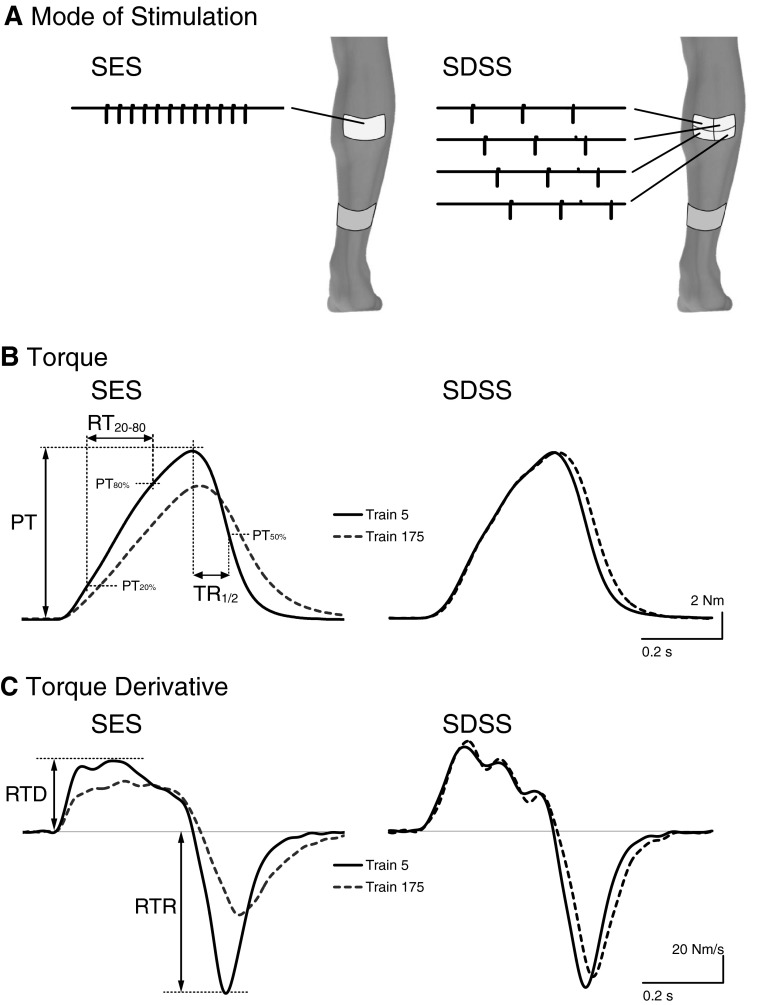
**a** Schematic representation of SES and SDSS electrodes placements. Stimulation pulse timing is also shown. Representative example of torque (**b**) and its derivative (**c**) for fifth train (*solid lines*) and 175th train (*dotted lines*) of fatiguing stimulation using SES and SDSS. PT: peak-to-peak torque amplitude; RT_20–80_: torque rise time between 20 % (PT_20 %_) and 80 % (PT_80 %_) of peak torque; TR½: half-relaxation time; *RTD* rate of torque development; *RTR* rate of torque relaxation

A programmable four-channel neuromuscular electrical stimulator (Compex Motion, Compex SA, Switzerland) was used to deliver transcutaneous electrical stimulation to the triceps surae muscle (TS). Self-adhesive gel electrodes (ValuTrode, Denmark) were placed over the proximal parts of the right gastrocnemius muscle (active electrode) and just above the Achilles tendon (reference electrode). Two modes of stimulation were compared: SDSS and SES (Fig. 1a). During SES, pulses were delivered conventionally through one active electrode at 40 Hz. Both active and reference electrodes were 9.0 × 5.0 cm. During SDSS, pulses were sequentially distributed among the arrayed active electrodes. One 9.0 × 5.0 cm electrode was used as a reference electrode in the same location as during SES, and four 4.5 × 2.5 cm electrodes were placed so that together they covered exactly the same area as the active electrode during SES. During SDSS, stimulation was delivered by sending a stimulation pulse to each of the four electrodes, one after another. Individual electrodes were stimulated at 10 Hz with a phase shift of 90° between successive electrodes, giving a resultant stimulation frequency of 40 Hz to the TS as a whole (Nguyen et al. 2011).

The stimulation current had a rectangular biphasic pulse waveform with a pulse duration of 300 μs. The stimulator was programmed to deliver a bout of fatiguing stimulation consisting of 180 trains which resulted in 180 muscle contractions. Each train of duration of 278.6 ms included 12 pulses; the trains were delivered with intervals between them of 733.4 ms. During each test, the stimulation was delivered for approximately 3 min.

Before each test, the stimulation intensity was increased in increments of 1 mA to determine the amplitude required to achieve an initial ankle torque at a level of about 8–12 N m across all participants. The selected initial torque values were sufficient to produce muscle fatigue, while not being too high to cause severe discomfort. For the participants in this study, the stimulation intensity was 41.7 ± 5.8 mA and 39.1 ± 5.6 mA for SES and SDSS, respectively (*p* = 0.044 by a paired *t* test).

Each test was conducted on a different day with at least 1 day of rest in between. Electrode positions were marked with a permanent marker to ensure that electrode placement was identical across tests.

#### Experimental setup and analysis

During the experiments, the participants were seated in an adjustable chair with the right foot firmly strapped to a device that measured isometric ankle torque in the sagittal plane using a reaction torque transducer (TS11, Interface, Inc., Scottsdale, AZ, USA). The positions of the hip and knee joints were set to 90° of flexion and that of the ankle joints to neutral position (0° dorsi-/plantar flexion).

We identified eight variables of interest. To indicate muscle force decay (Shields and Dudley-Javoroski 2006; Enoka and Stuart 1992; Thomas et al. 2003) during the fatiguing stimulation, we calculated (a) peak torque amplitude (PT) (Fig. 1b), (b) fatigue index (FI) and (c) torque–time integral (TTI). PT was defined as the peak-to-peak amplitude achieved from the torque–time series. FI was defined as the ratio between the minimum value among the maximal torques during the last five stimulus trains at the end of the 3-min stimulation and the maximum value during the initial five stimulus trains at the beginning of stimulation. Higher values indicate greater resistance to fatigue. TTI was defined as the integrated area under the torque–time curve through the whole bout of the fatigue protocol. To display and analyze the data, we used eight windows of five trains each, evenly spaced within a bout of 180 trains, and presented the mean values of those five trains (Shields and Dudley-Javoroski 2006).

To describe the muscle contraction progression and relaxation (Jones et al. 2006; Jones 2010; Bigland-Ritchie et al. 1983) during the fatiguing stimulation, we analyzed both ascending and descending phases of each muscle contraction. In the ascending phase, we calculated (d) torque rise time as the elapsed time in milliseconds between 20 and 80 % of peak torque for each train (RT_20–80_) (Fig. 1b) and (e) the rate of torque development (RTD) (Fig. 1c) as the maximal value of the first derivative of the torque signal. During the descending phase, we calculated (f) the half-relaxation time (TR½) (Fig. 1b) as the time it took for the torque to drop to one-half of its value, as well as (g) the rate of torque relaxation (RTR) (Fig. 1c) as the most negative value of the first derivative of the torque signal.

In addition, we characterized (h) the degree of contractile fusion during the ascending phase of the torque peaks by analyzing the percentage of the integrated power of the spectrum region within 8–12 Hz compared to the total power integral of the entire spectrum (Power_8–12Hz_). The range of 8–12 Hz was selected based on preliminary observations and due to the fact that the average firing rate of the plantar flexor muscles’ motor units is about 10 Hz (Bellemare et al. 1983; Bickel et al. 2011). Higher values of the Power_8–12Hz_ indicate less fusion during contraction.

#### Statistics

A two-way ANOVA with repeated measures and a post hoc Bonferroni test were applied to the group data (except of FI and TTI) to identify significant differences in the analyzed parameters between SDSS and SES, and throughout the time course of each fatiguing stimulation session. For FI and TTI measurements, *t* test comparisons were made to decompose significant effects after the fatiguing stimulation session (*α* = 0.05). Results of the group data are presented as mean values ± standard deviations.

### Experiment 2

#### Subjects

Tests were conducted in ten able-bodied participants (seven male, three female) aged 29.0 ± 7.0 years, with height of 176.6 ± 7.0 cm and weight of 73.4 ± 8.9 kg. Each participant gave written informed consent to the experimental procedure. This study was approved by the local ethics committee in accordance with the Declaration of Helsinki on the use of human subjects in experiments.

#### Transcutaneous neuromuscular electrical stimulation

The neuromuscular electrical stimulator, types and location of the stimulation electrodes were the same as during Experiment 1 for SDSS and SES. Ten stimulation pulses were delivered for each electrode at 1 Hz. For SDSS electrodes, pulses were delivered ten times in succession through each of the arrayed active electrodes [SDSS electrodes: medial proximal (MP) lateral proximal (LP), medial distal (MD), lateral distal (LD)] (cf. Fig. 6a). The stimulation current had a rectangular monopolar pulse waveform with a pulse duration of 1 ms. Before each test, the stimulation intensity was increased in increments of 1 mA to determine the amplitude required to achieve sufficient and well-differentiated muscle response, i.e., M-wave. For the participants in this study, the stimulation intensity was 42.1 ± 7.0 and 42.1 ± 7.0 mA for SES and SDSS, respectively (they were identical).

#### EMG recording

Surface EMG signals were recorded via four bipolar surface electrodes (Ag/AgCl, diameter of 9 mm, inter-electrode distance of 22 mm, Bortec Biomedical Ltd., Canada) placed at approximately equal distance from each other on the right soleus muscle, from its medial to lateral portions, distal to the belly of the medial gastrocnemius (EMG electrodes: M2, M1, L1, L2) (cf. Fig. 6a). A reference electrode (Ag/AgCl, diameter of 7 mm, Bortec Kendall Medi-Trace, Canada) was placed over the medial malleolus. The EMG signals were amplified and acquired using an AMT-8 EMG system (Bortec Biomedical Ltd., Canada) with a frequency bandwidth of 10–1,000 Hz and a common mode rejection ratio of 115 dB at 60 Hz. Finally, the EMG data of the right soleus were digitized at a sampling rate of 10 kHz using a data acquisition system (PowerLab 16/35, ADInstruments, Australia).

#### Experimental setup and analysis

The participants were positioned in the same setup as during Experiment 1. The magnitudes of the M-wave responses were calculated automatically by measuring the peak-to-peak amplitude of each response in the time interval of 10–20 ms after the onset of the stimulation pulse. Calculations were manually verified for all M-waves. The amplitude of the M-waves recorded in each of the four locations during SDSS was then normalized to the amplitude of the M-wave during SES. A one-way ANOVA with repeated measures was applied to the grouped data to identify significant differences in the magnitude of the M-wave responses from four EMG electrodes during the stimulation delivered through each of four SDSS electrodes. Results of the grouped data are presented as mean values ± standard deviations.

## Results

### Experiment 1

An example of the torque–time series at the beginning (train 5) and at the end (train 175) of the fatiguing stimulation during SES and SDSS protocols appears in Fig. 1b. The figure suggests that the participant’s performance was better maintained using SDSS than using SES. Figure 1c shows that the first derivative of the torque reveals unfused ascending phases of the muscle contractions with distinguishable peaks of approximately 10 Hz during both protocols. It can be seen that at the end of SES, the ascending phase becomes more fused, whereas at the end of SDSS the peaks are still well differentiated.

**Fig. 2:**
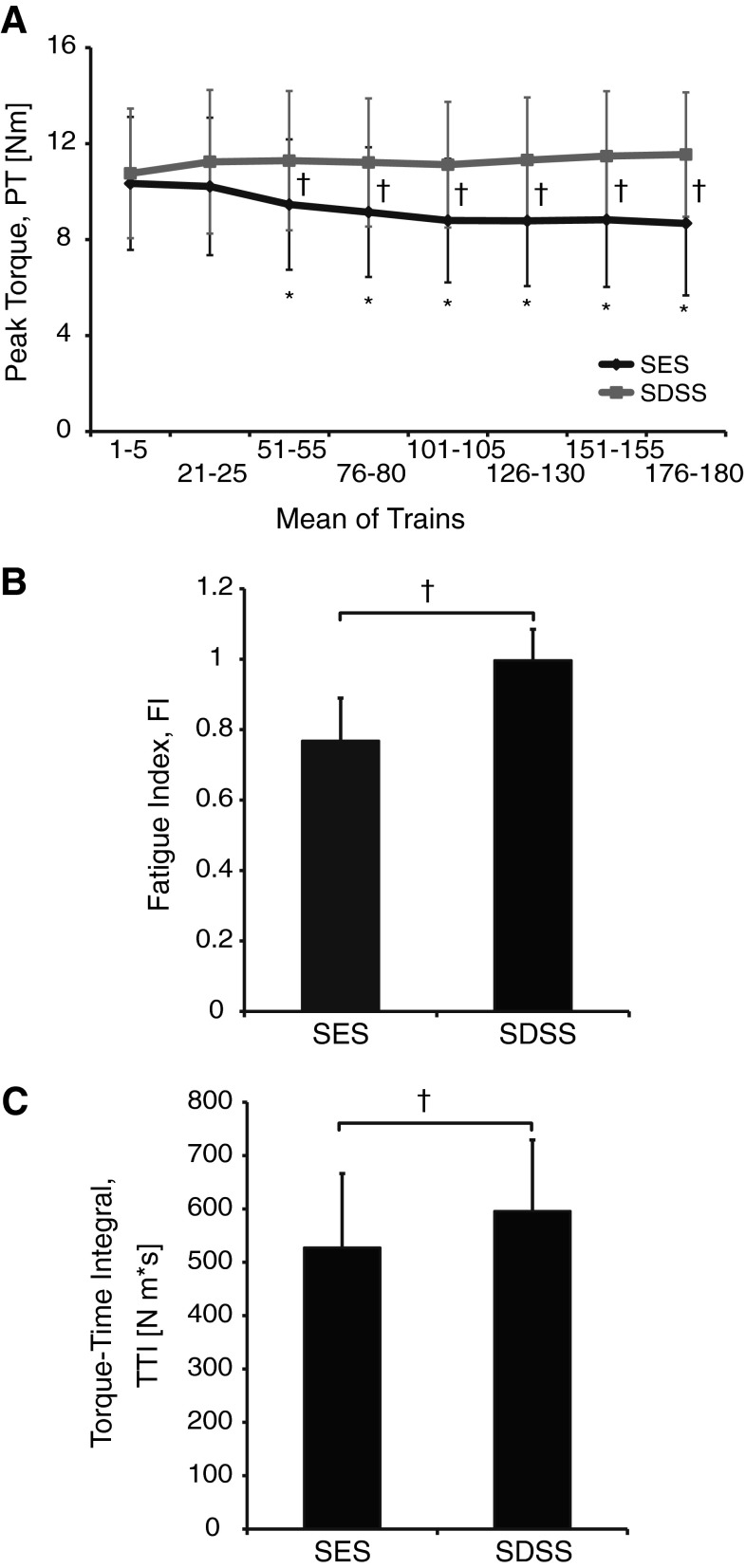
**a** Pooled effect of the fatiguing stimulation on the peak-to-peak torque amplitude (PT) values during SES (*black line*) and SDSS (*red line*). Mean values for five consecutive trains at eight points during the fatiguing stimulation are depicted. **b** Fatigue index (FI), and **c** torque–time integral (TTI) during SES and SDSS. *Significant differences between the initial values and those during the test; ^†^significant differences between the values during SES and SDSS (color figure online)

Figure 2 shows in detail the differences between the two types of stimulation in terms of the force decay measures. Figure 2a displays the pooled effect of the fatiguing stimulation on the PT values during SES and SDSS as a function of time. In the ANOVA results, significant main effects for the protocol (*F* = 44.0, *p* < 0.0001) and time (*F* = 4.29, *p* = 0.015), as well as the significant protocol by time interaction (*F* = 23.1, *p* < 0.0001), revealed that the muscle force decay developed significantly differently across time during SES and SDSS. The post hoc test revealed that, although the initial torque values were not different, the values significantly decreased relative to the first data bin starting at trains 51–55 (*p* < 0.0001) and remained reduced through the rest of the session (*p* < 0.0001) during SES. During SDSS, the torque values did not significantly differ from the initial value indicating that PT was maintained throughout the session. The FI (Fig. 2b) was 0.768 ± 0.121 and 0.997 ± 0.088 for SES and SDSS (*p* < 0.0001), respectively, demonstrating that SDSS resulted in significantly less fatigue than SES. The TTI (Fig. 2c) was 527 ± 139 and 596 ± 133 N m·s for SES and SDSS (*p* = 0.002), respectively.

**Fig. 3:**
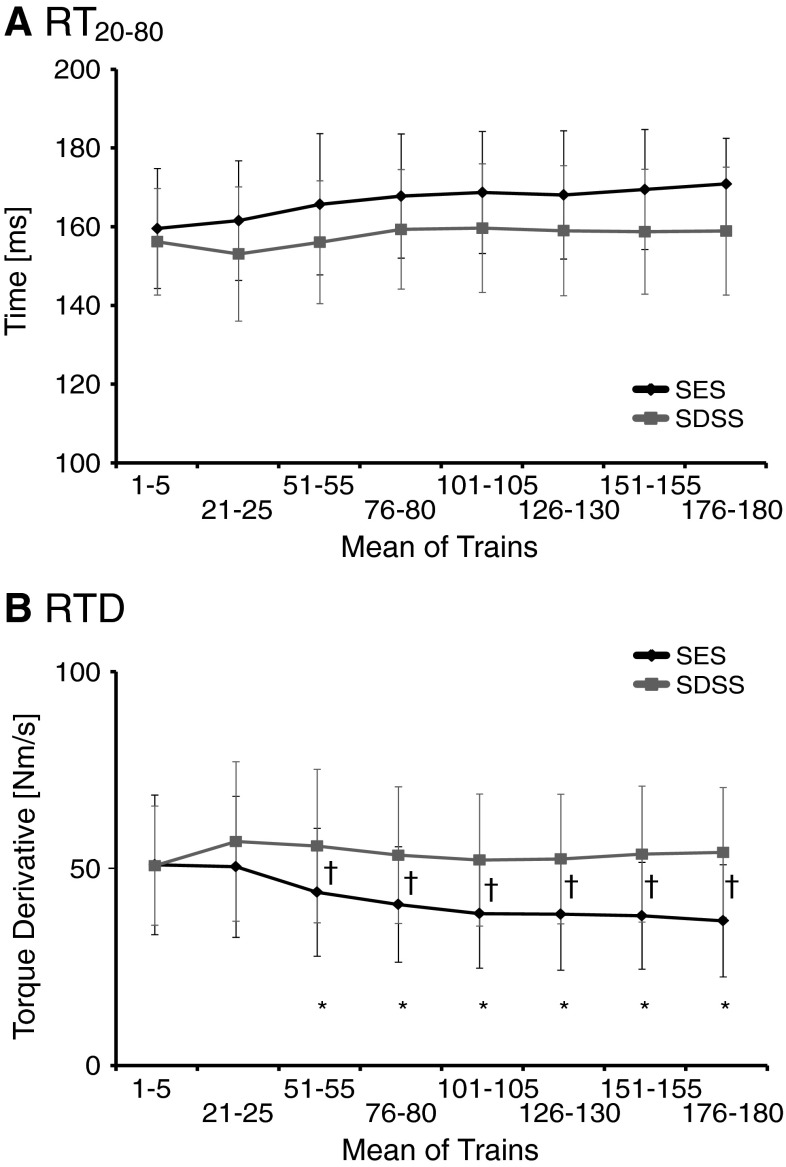
**a** Torque rise time (RT_20–80_), and **b** rate of torque development (RTD) during SES (*black line*) and SDSS (*red line*). *Significant differences between the initial values and those during the test; ^†^significant differences between the values during SES and SDSS (color figure online)

Figure 3 shows the dynamics of the ascending phase during SES and SDSS protocols. Figure 3a shows the RT_20–80_ across a selection of contractions during SES and SDSS protocols as a function of time. The ANOVA revealed significant main effects for the protocol (*F* = 5.69, *p* = 0.032), while the main effect of time (*F* = 3.080, *p* = 0.069) and the interaction (*F* = 1.185, *p* = 0.405) were not significant. The average duration of the contraction progression during SES protocol was longer than during SDSS: 166.4 ± 14.4 and 157.6 ± 14.8 ms, respectively (*p* = 0.03 by a paired *t* test). Figure 3b shows the results for RTD. The ANOVA of RTD yielded significant main effects for the protocol (*F* = 31.2, *p* < 0.0001) and time (*F* = 4.89, *p* = 0.02), as well as significant protocol by time interaction (*F* = 10.1, *p* = 0.002) indicating that the rate of torque development changed differently across time during SES and SDSS. The post hoc test revealed that the rate of torque development during SES decreased starting at trains 51–55 until the end of the fatiguing stimulation (*p* < 0.05) (Fig. 3b). The reduction of RTD was larger during SES than during SDSS as revealed by a significant difference between two protocols starting at trains 51–55 for the RTD (*p* < 0.05) (Fig. 3b).

**Fig. 4:**
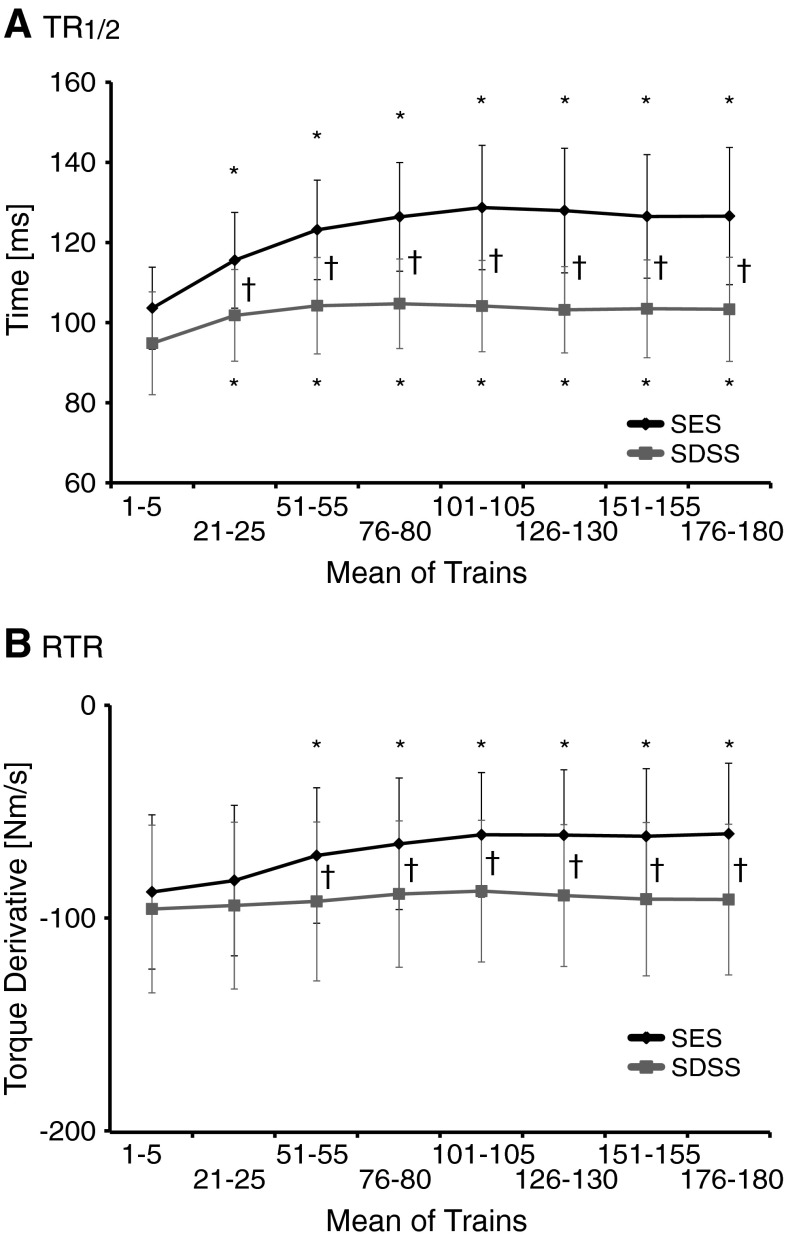
**a** Half-relaxation time (TR½) and **b** rate of torque relaxation (RTR) during SES (*black line*) and SDSS (*red line*). *Significant differences between the initial values and those during the tests; ^†^significant differences between the values during SES and SDSS (color figure online)

Figure 4 displays the progression of the relaxation phase during SES and SDSS protocols. For the TR½, the ANOVA revealed significant main effects for the protocol (*F* = 77.5, *p* < 0.0001) and time (*F* = 42.2, *p* < 0.0001), as well as protocol by time interaction (*F* = 8.99, *p* < 0.003) (Fig. 4a). The ANOVA of the RTR yielded significant main effects for the protocol (*F* = 63.9, *p* < 0.0001) and time (*F* = 4.76, *p* = 0.022), as well as the protocol by time interaction (*F* = 4.88, *p* = 0.02) (Fig. 4b). The post hoc test revealed that significant prolongation in muscle relaxation during the fatiguing stimulation occurred during SES, starting at trains 51–55 (*p* ≤ 0.001). The reduction of the rate of muscle relaxation was larger during SES than during SDSS as revealed by significant difference between two protocols starting at trains 21–25 for the TR½ (*p* = 0.0001) and at trains 51–55 for the RTR (*p* < 0.05).

**Fig. 5:**
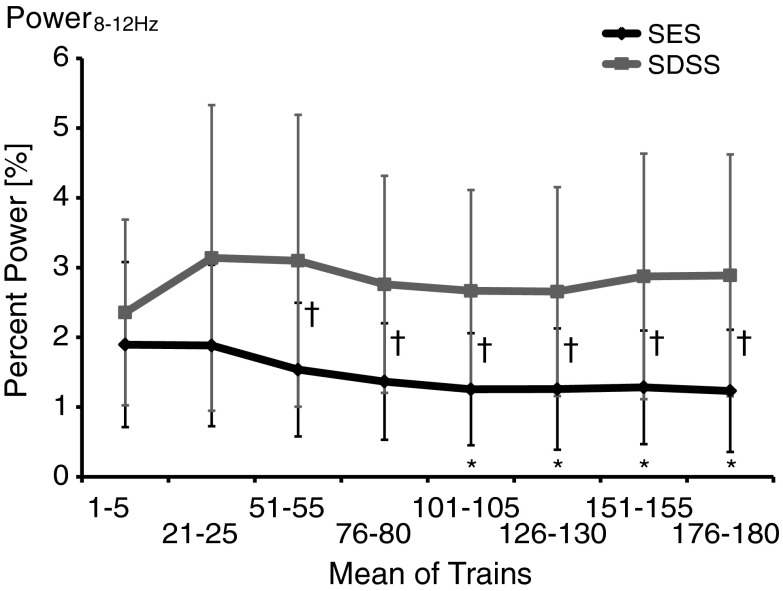
Percentage of the accumulated power of the spectrum within 8–12 Hz compared to outside of the frequency limit (Power_8–12Hz_). *Significant differences between the initial values and those during the test; ^†^significant differences between the values during SES and SDSS

Figure 5 shows the progression of Power_8–12Hz_ during SES and SDSS protocols. The analysis revealed significant main effects for the protocol (*F* = 25.8, *p* < 0.0001) and time (*F* = 5.28, *p* = 0.009), as well as the protocol by time interaction (*F* = 6.84, *p* = 0.002). The post hoc test revealed that, during SES, the Power_8-12Hz_ decreased starting at trains 101–105 (*p* ≤ 0.002), whereas during SDSS the Power_8-12Hz_ was maintained over the time course.

### Experiment 2

**Fig. 6:**
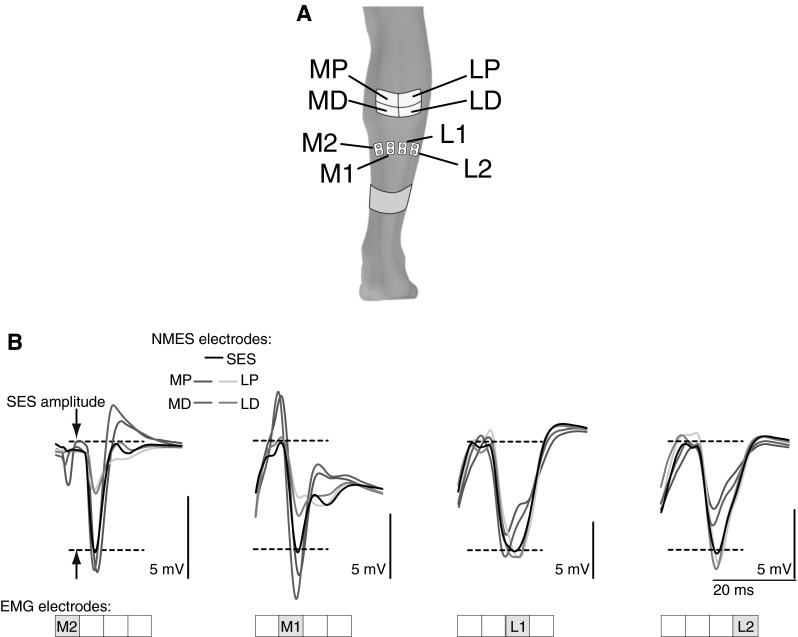
**a** Schematic representation of SDSS and EMG electrodes placements in Experiment 2. For SDSS electrodes, *MP* medial proximal; *LP* lateral proximal; *MD* medial distal; *LD* lateral distal. EMG electrodes placed over the m. soleus from medial to lateral portions as M2, M1, L1, and L2. **b** M-waves in each active electrode location (i.e., M2, M1, L1, and L2) during SDSS (*colored lines*) and SES (*black lines*) in one participant. Waves from 5 ms after the stimulation onset are shown. *Dotted lines* indicate M-wave amplitude during SES (color figure online)

Figure 6b demonstrates M-waves recorded at each active electrode location during SDSS and SES in one participant. It can be seen that during stimulation of the medial portion of the soleus muscle (electrodes MP and MD), the amplitude of the M-wave was larger under the medial EMG electrodes (M2 and M1) and smaller under lateral EMG electrodes (L2 and L1), whereas during stimulation of the lateral portion (electrodes LP and LD) the amplitude of the M-wave was larger under the lateral EMG electrodes and smaller under medial EMG electrodes.

**Fig. 7:**
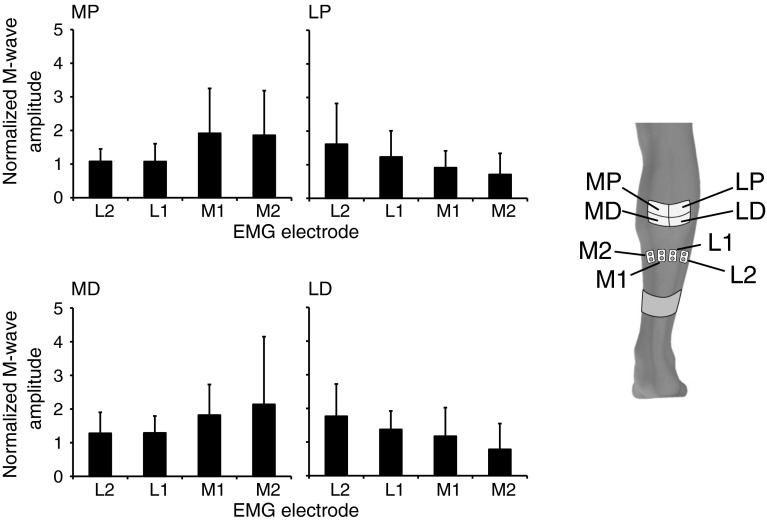
Grouped data of the normalized amplitude of the M-waves from each of the four muscle portions dependent on the location of the stimulation during SDSS (data normalized to SES). For SDSS electrodes, *MP* medial proximal; *LP* lateral proximal; *MD* medial distal; *LD* lateral distal. EMG electrodes placed over the m. soleus from medial to lateral portions as M2, M1, L1, and L2

The ANOVA of the grouped data revealed significant main effects of the amplitude of the M-waves from each of the three muscle portions dependent on the location of the stimulation MP, LP, MD, and LD: *p* = 0.03, *p* = 0.04, *p* = 0.06, and *p* = 0.008, respectively (Fig. 7).

## Discussion

We investigated the effectiveness of SDSS in reducing muscle fatigue during electrical stimulation in plantar flexors in an able-bodied population. Using small electrodes distributed over a relatively narrow area, we demonstrated no force decay and minor change in the muscle relaxation time during the fatiguing stimulation, as opposed to the more conventional approach of SES. In the series of additional experiments we demonstrated that during SDSS, the amplitude of the M-waves at different muscle portions depends on the location of the stimulation electrodes.

### Torque development

The analysis of the PT demonstrated that the peak torque rapidly decreased and remained depressed for the duration of the test during SES (Fig. 2a). In contrast, the PT values did not change during SDSS indicating that the torque was maintained at values close to the initial throughout the test. The FI was significantly reduced during SES indicating decrement of torque, whereas during SDSS the FI was close to 1, indicating higher ability to maintain the torque in the course of the stimulation (Fig. 2b). The TTI that has been shown depends not only on the peak torque, but also on the contractile properties and the degree of fusion between the individual summated twitch responses (Shields and Dudley-Javoroski 2006). The TTI was greater during SDSS than during SES (Fig. 2c), indicating that a combination of higher torque-producing capacity and reduced fatigability during SDSS enabled it to perform a greater magnitude of contractile work during repetitive activation when compared with SES. Likewise, using similar measures describing the muscle force decay during the fatiguing stimulation (e.g., peak torque amplitude, fatigue index, and torque–time integral) to ones in our previous study (Nguyen et al. 2011), we verified and extended these previous results obtained in one individual with spinal cord injury and demonstrated the higher ability to maintain torque longer during SDSS than during SES for a group of able-bodied individuals. Thus, these results suggest, in terms of torque decrement, that muscle fatigue occurred during SES, while it did not occur during SDSS under the applied stimulation condition.

As mentioned above, the PT did not change during SDSS (Fig. 2a) and the FI was close to 1, i.e., the FI was not statistically different from 1 (0.997 ± 0.088, *p* = 0.881 by *t* test), indicating that torque was maintained during SDSS. This is different from our previous study (Nguyen et al. 2011), where the torque decreased immediately after onset of stimulation during both SES and SDSS. This resulted in a much larger value of FI during SDSS in the present (Fig. 2b) than in our previous study (i.e., 0.458 ± 0.074). This discrepancy can be explained by the difference in the fatiguing stimulation protocol: intermittent stimulation in the present study as opposed to continuous stimulation in the previous study. In addition, the previous experiment was performed in an individual with complete SCI, which can cause the observed difference in torque development. Changes in paralyzed muscle include decreased oxidative enzymes (Grimby et al. 1976), decreased cross-sectional area (Castro et al. 1999), impaired excitation–contraction coupling (Talmadge et al. 2002; Shields 1995), and transformation to a fast-fatigable phenotype (Crameri et al. 2000; Grimby et al. 1976; Shields 1995). These factors can result in less fatigue resistance after SCI (Bickel et al. 2011; Shields 1995, 2002; Slade et al. 2003; Thomas et al. 2003) compared to able-bodied participants.

### Muscle contractile properties

Muscular fatigue is characterized not only by loss of muscle force, but also by change of muscle contractile properties such as a slowing of the muscle contraction progression (Bigland-Ritchie et al. 1983). The slowing of contraction progression is associated with decrease in the rate of calcium transport and impairment of cross-bridge attachment–detachment rates (Shields and Dudley-Javoroski 2006; Jones et al. 2006), which has been suggested to play a major part in the loss of power associated with muscle fatigue. In measuring the muscle contractile properties, it has been noted previously by Bigland-Ritchie et al. (Bigland-Ritchie et al. 1983) that any indirect measurement of muscle contractile properties is often subject to errors when made from intact human subjects. Therefore, a more detailed and multifaceted analysis was required, including the rates of muscle contraction progression and relaxation in addition to torque rise time and half-relaxation time.

The results demonstrated that the RT_20–80_ values were significantly larger during SES than during SDSS, while the RT_20–80_ values were maintained during periods of fatiguing stimulation (Fig. 3a). Given the continued decline of torque in the course of the fatiguing stimulation during SES (e.g., Fig. 2a), the increased rise time quantified by RT_20–80_ would then implicate reduced contraction progression. This suggestion was confirmed by the differences in the RTD during the two protocols: RTD decreased within 1 min of the fatiguing stimulation during SES, whereas RTD was maintained during SDSS (Fig. 3b). Taken together, these findings suggest that the muscle contraction progression was slowed down during SES, while it was maintained during SDSS.

In addition to a slowing of the muscle contraction progression, it has been shown that slowing in relaxation is a characteristic feature of muscle that has been fatigued by metabolically demanding contractile activity (Edwards et al. 1972, 1975; Cady et al. 1989; Westerblad and Lannergren 1991). Our results on RTR clearly suggest that muscle relaxation was slowed down during SES, whereas it was maintained during SDSS (Fig. 4b). These results are in line with the above-mentioned findings regarding muscle contraction progression, suggesting that muscle fatigue in terms of muscle contractile properties occurred during SES, whereas it was not the case during SDSS. However, the other measure for the relaxation of muscle contraction, i.e., TR½, indicates a somewhat different finding (Fig. 4a). The result of TR½ showed a similar finding as above in that the relaxation of muscle contraction was slowed down during SES, but it showed that it slowed down during SDSS as well. This result suggests that TR½ captured a slight sign of muscle fatigue during SDSS.

Further evidence for an association between muscle contractile properties and fatigue can be seen in the results for contractile fusion, i.e., Power_8–12Hz_ (Fig. 5). Slowing in the contraction progression inevitably results in greater within-train torque summation by limiting the amount of relaxation that can occur before the arrival of the subsequent stimulus pulse (Shields and Dudley-Javoroski 2006). Indeed, a previous study reported that increased fusion of the tetanus torque occurred due to muscle fatigue (Shields et al. 1997). The results of Power_8-12Hz_ indicated that, in later periods of fatiguing stimulation, it decreased during SES, while it was maintained during SDSS. These results suggest that the degree of fusion increased during SES, but not during SDSS. Therefore, in later periods of fatiguing stimulation, the difference in degree of fusion between SES and SDSS became more prominent, i.e., the degree of fusion became larger in SES than in SDSS. The stimulation frequencies during SES, i.e., 40 Hz, were much higher than the physiologic firing rate of human plantar flexors, which is about 10 Hz (Bellemare et al. 1983; Dalton et al. 2009) that corresponds to the stimulation frequency for each electrode during SDSS. Therefore, each subcompartment of muscle might be activated at its physiologic firing rate during SDSS and all muscle fibres were activated at a very high frequency during SES, resulting in a larger degree of fusion during SES than during SDSS.

### Possible mechanisms

In our previous study (Nguyen et al. 2011), we speculated that the mechanism for the effectiveness of SDSS was that different sets of muscle fibers were activated by each of the four electrodes during SDSS. Our current findings in experiment 2 agree with this speculation. We demonstrated that during SDSS, the magnitude of the muscle response in different portions of the muscle depends on the location of the stimulation except for MD stimulation (Figs. 6b, 7). In the case of MD stimulation, the difference was pronounced and the *p* value was very close to statistical significance (*p* = 0.06). That is, the sequentially distributed electrodes eventually activated different parts (branches) of the motor nerve and, as a consequence, different subcomponents of soleus muscle.

According to this finding, different subcomponents of muscles must be activated at a frequency of 10 Hz. Since this frequency for each subcomponent during SDSS is much slower than a frequency of 40 Hz for the corresponding subcomponent during SES, the muscle fatigue must be much less at each subcomponent as the increased time between subsequent activation of motor units allows greater recovery (Marsden et al. 1983). Additionally, as the frequency of 10 Hz is the natural firing frequency of soleus (Bellemare et al. 1983; Dalton et al. 2009), this may contribute to the effectiveness of SDSS in preventing muscle fatigue.

## Conclusion

The present work demonstrated that torque decay was negligible during the fatiguing stimulation using spatially distributed sequential stimulation in plantar flexor muscles for able-bodied individuals, whereas there was a marked torque decay during single active electrode stimulation. In addition, we demonstrated that the spatially distributed sequential stimulation did not affect much the muscle contractile properties during the fatiguing stimulation, whereas single active electrode stimulation slowed down muscle contraction progression and relaxation. These results suggest that muscle fatigue was negligible during spatially distributed sequential stimulation, but marked during single active electrode stimulation. Further, we demonstrated that during the spatially distributed sequential stimulation, the amplitude of the M-waves at each muscle portion is dependent on the location of the stimulation electrodes, suggesting that different sets of muscle fibers are activated alternatively by different electrodes, which is closer to physiological activation. We conclude that because of this, spatially distributed sequential stimulation is more effective in reducing muscle fatigue compared to single active electrode stimulation, which must have a prominent advantage in neurorehabilitation using transcutaneous neuromuscular electrical stimulation.
